# Endocrinological Evaluations of a Neurofibromatosis Type 1 Cohort: Is it Necessary to Evaluate Autoimmune Thyroiditis in Neurofibromatosis Type 1?

**DOI:** 10.4274/balkanmedj.2015.1717

**Published:** 2017-12-01

**Authors:** Serhat Güler, Gözde Yeşil, Hasan Önal

**Affiliations:** 1 Clinic of Pediatric Neurology, Edirne Sultan 1st Murat State Hospital, Edirne, Turkey; 2 Department of Medical Genetics, Bezmialem Vakıf University School of Medicine, İstanbul, Turkey; 3 Clinic of Pediatric Endocrinology and Metabolic Diseases, Kanuni Sultan Süleyman Training and Research Hospital, İstanbul, Turkey

**Keywords:** Neurofibromatosis 1, thyroid hormones, autoimmune thyroiditis, anti-thyroglobulin antibody, anti-thyroid peroxidase antibody

## Abstract

**Background:**

Neurofibromatosis type 1 is an autosomal dominant neurocutaneous disorder in which the coexistence of autoimmune thyroiditis and thyroid gland tumours has been reported previously.

**Aims:**

To determine the thyroid function and autoimmune thyroid diseases in neurofibromatosis type 1 patients in order to identify the possible association between neurofibromatosis type 1 and thyroid diseases.

**Study Design:**

Case-control study.

**Methods:**

The study includes 78 consecutive patients diagnosed with neurofibromatosis type 1 between June 2010 and June 2014 and 50 healthy controls. Baseline demographic data were generated from patient examination record forms, including age, sex, height, and weight, as well as levels of free triiodothyronine, free thyroxine, thyroid-stimulating hormone, anti-thyroid peroxidase and anti-thyroglobulin levels.

**Results:**

Mean age, sex, and body mass index were similar in both groups (p>0.05). The mean levels of free triiodothyronine, free thyroxine, and thyroid-stimulating hormone were not statistically different between the neurofibromatosis type 1 and control groups. Similarly, no statistically significant difference was observed between the neurofibromatosis type 1 and control groups for anti-thyroid peroxidase and anti-thyroglobulin positivity (2.5% vs 0%, p>0.05).

**Conclusion:**

Screening for autoimmune thyroid disease and thyroid function seems to be unnecessary in patients with neurofibromatosis type 1.

Neurofibromatosis type 1 (NF1) is an autosomal dominant disorder with an estimated prevalence of about 1 in 3.000. NF1 is caused by a NF1 gene mutation on chromosome 17q11.2 (2). The NF1 gene encodes neurofibromin, which acts as a tumour suppressor protein. The neurofibromin protein is thought to play a key role in autoimmune diseases and NF1 (17). Autoimmune thyroiditis (AT) is rarely seen in association with NF1. AT and neuroendocrine tumours of the thyroid gland have been reported in patients with NF1 but were mostly regarded as coincidental findings (3,4,5). No study has reported a relationship between NF1 and AT. In this study, we determined thyroid function and autoimmune thyroid diseases in patients with NF1 to identify the possible association between NF1 and thyroid disease.

## MATERIALS AND METHODS

The study included 78 consecutive patients with NF1 who were admitted to the departments of Paediatric Endocrinology and Metabolic Diseases of Kanuni Sultan Süleyman Training and Research Hospital and Medical Genetics of Bezmialem Vakıf University of Medicine from June 2010 to June 2014, along with 50 healthy controls. The patients with NF1 were diagnosed clinically based on the National Institutes of Health criteria (6). Informed, written consent was obtained from the parents of the children in the study. This study was approved by the local ethics committee of Bezmialem Vakıf University. Exclusion criteria were; previously known thyroid dysfunction or goitre and use of any drugs that could interfere with thyroid function prior to the study.

The physical examinations of all patients were performed by the same paediatric endocrinologist, and anthropometric measurements were recorded. Height was measured without shoes to the nearest 0.1 cm and weight was measured to the nearest 0.1 kg while the subjects wore light clothing using a combined stadiometer and calibrated electronic scale (Tess EBB; Çomak Tartı, İstanbul, Turkey). Body mass index (BMI) was calculated as follows: BMI= body weight (kg)/height^2^ (mm^2^). According to the Marshall and Tanner (7,8) developmental scales, the pubertal stages of girls and boys were defined by breast and pubic hair development and by gonadal stage and pubic hair development, respectively. The neck and thyroid gland were examined.

Blood specimens were collected between 9 and 10 am after an overnight fast. A 3 mL blood sample was taken, and centrifuged to obtain the plasma, which was stored at -20 °C until analysis. Free triiodothyronine (FT3) (normal range 3.0-9.0 pmol/L), free thyroxine (FT4) (normal range 8.5-40.0 pmol/L), thyroid-stimulating hormone (TSH) (normal range 0.8-5.4 mIU/L), anti-thyroid peroxidase (anti-TPO) (normal range 0-30 IU/L) and anti-thyroglobulin (anti-TG) (normal range 0-60 IU/L) levels were measured using the electrochemiluminometric assay method at the beginning of or during the study period (ADVIA Centaur XP Immunoassay System, Siemens, Erlangen, Germany). All patients underwent thyroid ultrasonographic examinations as a second step approach.

### Statistical analysis

The data were analysed using the SPSS version 20 statistical package program (SPSS, IBM, Chicago, IL, USA). A confidence interval of 95% and a two tailed p value ≤0.05 were considered statistically significant. For the primary outcome, allowing a type 1 (α) error of 0.05 and a sample size of 78 patients resulted in 0.80 power in this study. The numerical data were analysed with Kolmogorov-Smirnov test to determine whether the numerical variants were parametric. The parametric numerical data between the groups were compared with Student’s t-test, and all numerical data were noted as the mean ± standard deviation (SD). The nonparametric numerical data were evaluated with Mann Whitney-U tests. The categorical variables were assessed via χ^2^ tests and Fisher’s exact test was used if needed.

## RESULTS

The study included 78 patients diagnosed with NF1 [44 males (56.4%) and 34 (43.6%) females; mean age, 11.11±4.55 years; mean BMI, 20.05±3.22 kg/m^2^]. The control group included 50 healthy children [29 males (58%) and 21 (42%) females; mean age, 11.21±4.50 years; mean BMI, 19.84±3.25 kg/m^2^]. Mean age, sex and BMI were similar in both groups (p>0.05). FT3 (mean ± SD]) was 5.92±0.62 pmoI/L in the NF1 group and 6.02±0.56 pmoI/L in the control group (p=0.387). FT4 was 14.85±2.04 pmoI/L in the NF1 group and 15.18±1.65 pmoI/L in the control group (p=0.339). TSH was 2.90±2.61 mIU/L in the NF1 group and 2.93±1.12 mIU/L in the control group (p=0.944).

FT3, FT4 and TSH levels were compared between female and male patients. Mean FT3 (mean ± SD) was 5.99±0.62 pmoI/L in the male and 5.84±0.62 pmoI/L in the female NF1 group (p=0.278). FT4 was 14.82±1.96 pmoI/L in the male and 14.88±2.17 pmoI/L in the female NF1 group (p=0.902). TSH was 2.57±1.14 mIU/L in the male and 3.33±3.73 mIU/L in the female NF1 group (p=0.207).

The pubertal examination revealed that 30 patients were pre-pubertal and 48 patients were in different stages of puberty. Of the 50 control patients, 24 were pre-pubertal and 26 were pubertal (Table 1). Five patients in the NF1 group had (6.4%) goitre. Two of the 78 patients with NF1 were positive for the anti-TPO (normal, 0-30 IU/L) and anti-TG antibodies (normal, 0-60 IU/L) (2.5%). The first patient was a 12-year-old girl with 450 IU/L anti-TPO, 1.200 IU/L anti-TG, 0.78 mIU/L TSH and 20.82 kg/m^2^ BMI. The second patient was a 15-year-old girl with 750 IU/L anti-TPO, 524 IU/L anti-TG, 2.55 mIU/L TSH and 29.86 kg/m^2^ BMI. Thyroid ultrasonography of both patients showed an enlarged gland with a heterogeneous parenchyma (Table 2). Three of the five patients who had goitre were euthyroid. No thyroid autoantibodies were detected in the control group.

A 10-year-old girl in the NF1 group had a TSH level above the normal range indicating subclinical hypothyroidism (SH) (TSH, 22 mIU/L; normal, 0.8-5.4 mIU/L) (1.2%). Thyroid autoantibodies were normal (anti-TPO, <5 IU/L and anti-TG, <5 IU/L) (Table 2). She had no goitre and her BMI was 29.26 kg/m^2^. Thyroid ultrasound was normal in this patient. No SH was detected in the controls. The TSH levels in three of 78 patients with NF1 were below the normal range (3.8%) (Table 2) and ultrasonographic thyroid exams were normal. Thyroid autoantibodies of these three patients were in the normal ranges (anti-TPO, <5 IU/L and anti-TG, <5 IU/L). The TSH levels of all controls were in the normal range.

In total, thyroid function tests were abnormal in three (3.8%) of 78 patients with NF1 (1.3% SH, 2.5% AT); all three patients were female and were in various stages of puberty (Table 2). In addition, thyroid nodules were detected in two patients in the NF1 group and in one control; both had normal thyroid function tests and examinations.

Our patients with NF1 had other endocrinological conditions, such as obesity (n=7), hirsutism (n=2), short stature (n=18), precocious puberty (n=6) and gynaecomastia (n=2).

## DISCUSSION

The concomitant occurrence of autoimmune thyroid diseases [Hashimoto’s thyroiditis (HT) and Graves’ disease], and thyroid neuroendocrine tumours and other neoplasms (C-cell hyperplasia, medullary thyroid cancer, papillary thyroid cancer and neurofibroma) with NF1 has been reported in previous studies (4,9,10,11,12,13). The present study concluded that the comorbidity of AT with NF1 in children and adolescents is uncommon. This is the first report that has searched for autoimmune thyroid diseases in patients with NF1.

Neurofibromin is the protein product of the NF1 gene and is a GTPase-activating protein that down-regulates the cellular p21-ras proto-oncogene. Loss of function of neurofibromin may lead to uncontrolled cell growth and increased tumour formation in patients with NF1 (3). In addition, abnormal neurofibromin production suppresses Fas ligand expression, therefore preventing the apoptosis of CD4+ T cells, which is important for the development of autoimmunity (14).

HT is rarely associated with NF1, and only five cases have been reported so far (3,9,15,16,17). Initially, Yalcin et al. (3) described HT and vitiligo in a 20-year-old female patient diagnosed with NF1 and Noonan syndrome. Nanda (15) emphasised the relationship between NF1 and autoimmune diseases. In that report, one patient had vitiligo, whereas the other had alopecia areata and HT. Nabi (9) described a 30-year-old woman with the concomitant occurrence of goitre and NF1. Sasazawa et al. (16) reported a 51-year-old patient with NF1 and HT who suffered a coma as a cause of mixedema. Faraz et al. (17) presented a 53-year-old woman with concomitant high blood pressure and NF1. The prevalence of HT in the general population is 2% and it is more common in females (18). There was no statistically significant difference in thyroid function between male and female NF1 patients. In the present study, two of the 78 patients with NF1 were diagnosed with HT at a frequency of 2.5%; both were asymptomatic and euthyroid. Goitre was found on a thyroid gland examination in both patients. Ultrasonography revealed an enlarged thyroid gland with a heterogeneous parenchyma.

Graves’ disease is another autoimmune thyroid disease that occurs in patients with NF1. To date, three patients with NF1 have been diagnosed with Graves’ disease (10,19,20). However, none of our patients had Graves’ disease. AT cases in patients with NF1 have all presented as incidental case reports. Despite the pathophysiological effect of neurofibromin on autoimmunity, this study showed that the frequency of AT in patients with NF1 and the general population does not differ significantly. Our study revealed that thyroid dysfunction was prominent in females (Table 2).

SH is described as a mild elevation of TSH levels (5-25 mIU/L) in the presence of normal thyroid hormone concentrations (21). SH has a benign and remitting course with a low risk of progression to overt hypothyroidism and is known to have no effects on neuropsychological function. SH prevalence in the paediatric population is reported to be slight <2% (22). In the present study, one female patient had SH (1.2%); SH was not detected in any of the controls (Table 2). This is the first patient with coexisting NF1 and SH but the result may be due to the lack of investigation into thyroid function in patients with NF1. SH is a frequent entity in the obese population. A paediatric study reported that 28.4% of patients with SH were also obese or overweight (23). Seven obese patients were in our cohort; only one patient had SH and one had HT. The remaining five patients had normal thyroid function.

Short stature is a common problem in children and young adults with NF1. Studies in patients with NF1 show that 13-33% of affected individuals are shorter than expected (24,25). This rate was 23.1% in the NF1 group in the present study. One of three patients with NF1, who had abnormal thyroid tests, was also short (<3rd percentile) (Table 2). Hegedus et al. (26) reported that neurofibromin affects the hypothalamo-hypophyseal axis and that growth hormone and insulin-like growth factor levels decrease. They also stated that short stature is caused by suppression of the hypothalamo-hypophyseal axis. Besides obesity and short stature, other endocrinological symptoms observed in our study were precocious puberty, hirsutism and gynaecomastia.

In our cohort, low TSH levels were detected in three patients with NF1 (3.8%). However, none of these patients had signs of goitre on ultrasonography and were free of autoantibodies. TSH levels of all controls were in the normal range. According to these findings, we propose that low TSH values may reflect an impaired hypothalamo-hypophyseal-thyroid axis. However, no study has explored how the hypothalamo-hypophyseal-thyroid axis is affected in patients with NF1. TSH values of the three patients returned to normal after 1 year.

The prevalence of AT was 2.5% in our patients with NF1. This study showed that the frequency of AT in the NF1 and general populations did not differ significantly; however, it was high compared with the control group. The limitation of the present study was the small number of patients and the control group. There is a need for a large scale and prospective study to confirm these results.

The association between NF1 and AT may have been a coincidence in our cohort. Our results suggest that screening for autoimmune thyroid disease and thyroid function is unnecessary. A thyroid gland examination is of the greatest value as the first step in an investigation, and thyroid hormone levels and ultrasonography should be performed as a second step in cases of suspicious findings.

## Figures and Tables

**Table 1:**
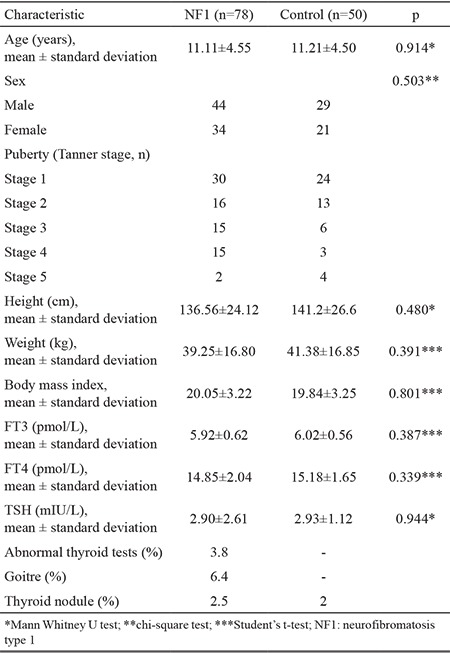
Clinical characteristics and thyroid hormone levels in NF1 patients

**Table 2:**
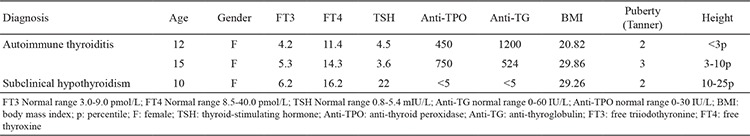
The characteristics of three patients with abnormal laboratory findings
